# ADN tumoral circulante en pacientes con cáncer: perspectivas desde el laboratorio clínico

**DOI:** 10.1515/almed-2025-0093

**Published:** 2025-08-19

**Authors:** Francisco J. Illana, Esther Fernández-Galán, José Luis Muñoz-Bravo, Laura Valiña Amado, Carme García Martín, Carolina González-Fernández, Sílvia Miró-Cañís, Jaume Trapé, Antonio Martínez-Peinado, Xavier Filella, Alvaro González, Antonio Barco Sánchez, Angel Díaz-Lagares, en nombre de la Comisión de Marcadores Biológicos del Cáncer de la Sociedad Española de Medicina de Laboratorio

**Affiliations:** Servicio de Bioquímica, Hospital de la Santa Creu i Sant Pau, IIB Sant Pau, Barcelona, España; Servicio de Bioquímica y Genética Molecular, CDB, Hospital Clínic de Barcelona, IDIBAPS, Universidad de Barcelona, Barcelona, España; Servicio de Análisis Clínicos, Hospital General Universitario de Elche, Elche, España; Fundación para el Fomento de la Investigación Sanitaria y Biomédica de la Comunidad Valenciana (FISABIO), Elche, España; Departamento de Medicina de Laboratorio, Hospital Universitario Son Espases, Palma, España; Grupo de Terapias Avanzadas y Biomarcadores en Oncología Médica, Institut d’Investigació Sanitària de les Illes Balears (IdISBa), Palma, España; Departamento de Bioquímica e Inmunoensayos, Laboratorio de Análisis Clínicos, Hospital Universitario Germans Trias i Pujol, Badalona, España; Departamento de Medicina de Laboratorio, Althaia Xarxa Assistencial Universitària de Manresa, Manresa, Cataluña, España; Laboratorio de Análisis Clínicos, CLILAB Diagnòstics, Vilafranca del Penedès, España; Unidad de Gestión de Análisis Clínicos, Sección de Genética Molecular, Hospital Universitario Reina Sofía, Córdoba, España; Departamento de Bioquímica Clínica, Universidad de Navarra, Pamplona, España; Departamento de Bioquímica Clínica, Hospital Universitario Virgen Macarena, Sevilla, España; Servicio de Análisis Clínicos, Complejo Hospitalario Universitario de Santiago (CHUS), Santiago de Compostela, España; Unidad de Epigenómica, Epigenómica del Cáncer, Oncología Médica Traslacional (ONCOMET), Instituto de Investigación Sanitaria de Santiago de Compostela (IDIS), Santiago de Compostela, España; Centro de Investigación Biomédica en Red Cáncer (CIBERONC), ISCIII, Madrid, España

**Keywords:** biomarcadores, cáncer, ctDNA, biopsia líquida, variantes genéticas somáticas

## Abstract

El análisis de ADN tumoral circulante (ctDNA) es un método no invasivo de gran relevancia para la caracterización molecular de los tumores sólidos, que aporta información valiosa sobre el perfil genético del cáncer. Actualmente, el análisis de mutaciones somáticas del ctDNA se emplea en la clínica para la selección de terapias dirigidas en enfermedades oncológicas avanzadas. Avances recientes también han revelado su potencial en la detección precoz, el pronóstico y la evaluación de enfermedad mínima residual, así como en la predicción y el seguimiento de la respuesta a terapia. En los últimos años, se han logrado importantes avances en el desarrollo de diversos métodos basados tanto en la secuenciación de nueva generación (NGS, por sus siglas en inglés) como en la realización de PCR para detectar variantes genéticas en el ctDNA de los pacientes oncológicos. Sin embargo, a pesar de las novedosas oportunidades que brinda el análisis de ctDNA, este aún presenta algunas limitaciones. La falta de normalización de los protocolos preanalíticos y analíticos y de la interpretación de los resultados, así como la heterogeneidad en la sensibilidad de las pruebas siguen siendo una asignatura pendiente que deberá ser abordada antes de impulsar un uso generalizado de las pruebas de ctDNA en la clínica. Además de las mutaciones somáticas, se están obteniendo resultados prometedores en estudios sobre la metilación del ADN (epigenómica) y el tamaño de los fragmentos de ADN (fragmentómica) en diversos tipos de fluidos biológicos como biomarcadores no invasivos para el manejo del cáncer. En la presente revisión, se describen las aplicaciones clínicas de las variantes genéticas somáticas en el ctDNA, haciendo hincapié en los biomarcadores del cáncer, y enumerando los factores esenciales para lograr una implementación eficaz en el laboratorio clínico y en el manejo de las enfermedades oncológicas.

## Introducción

**Figura 1:**
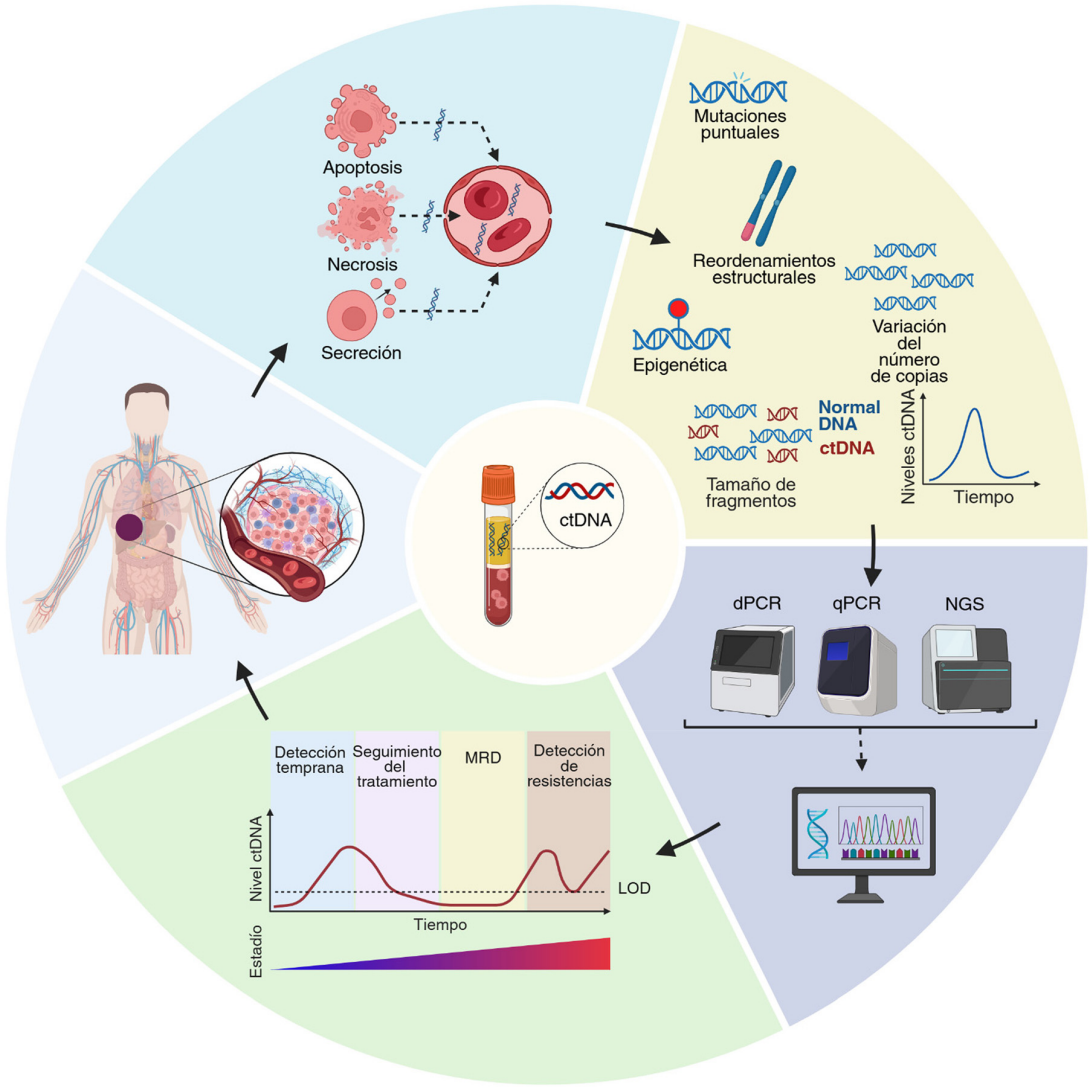
Representación esquemática del análisis de ctDNA en pacientes con cáncer. Los tumores sólidos liberan ctDNA a la circulación (por apoptosis, necrosis o secreción), el cual contiene alteraciones genéticas y epigenéticas específicas representativas del perfil molecular tumoral. Los fragmentos de ADN en circulación permiten la evaluación potencial de distintas características del ctDNA, incluyendo mutaciones puntuales, reordenamientos estructurales, variaciones en el número de copias, epigenética (metilación del ADN), tamaño de los fragmentos y niveles de ctDNA. Los ensayos moleculares actuales para el estudio del ctDNA permiten analizar alteraciones genéticas somáticas específicas mediante dPCR o qPCR, o realizar genotipado molecular con tecnologías basadas en NGS. Las posibles aplicaciones clínicas en oncología incluyen cribado/detección precoz del cáncer, monitorización del tratamiento, detección de enfermedad mínima residual (MRD) y detección de resistencias. ctDNA: ADN tumoral circulante; dPCR: PCR digital; qPCR: PCR cuantitativa; NGS: Secuenciación de nueva generación; LOD: Límite de detección; MRD: Enfermedad mínima residual. Figura creada con BioRender (https://BioRender.com/x96k221).

En los últimos años, se ha desarrollado la biopsia líquida como método no invasivo para la caracterización molecular de los tumores mediante el análisis de los componentes tumorales circulantes en distintos fluidos biológicos, principalmente el plasma (Figura 1) [1], 2]. Esta nueva estrategia alberga un enorme potencial en el manejo de las enfermedades oncológicas, con aplicaciones en el cribado y la detección precoz, el pronóstico, la detección de enfermedad mínima residual (MRD, por sus siglas en inglés) y el seguimiento de la respuesta a tratamiento [1], 3]. Entre los componentes analizados, el ADN tumoral circulante (ctDNA, por sus siglas en inglés) destaca por su capacidad para detectar mutaciones somáticas, proporcionando información clínica de gran relevancia [4]. Aunque las mutaciones somáticas son los biomarcadores moleculares más avanzados empleados actualmente en la clínica, se están obteniendo resultados prometedores con otras características del ctDNA, como la metilación del ADN [5] y los perfiles del tamaño de los fragmentos [6]. Avances recientes en las pruebas de ctDNA han demostrado su potencial para orientar las terapias dirigidas en el cáncer avanzado, existiendo evidencia creciente sobre su aplicabilidad en la práctica clínica. Sin embargo, quedan algunas dificultades por superar, como la normalización de los procedimientos preanalíticos y analíticos y la interpretación de los resultados [4]. En la presente revisión, se describen las aplicaciones clínicas del ctDNA, enfatizando su potencial como biomarcador en las enfermedades oncológicas. Así mismo, se abordan los factores preanalíticos y analíticos, las pruebas de detección y la interpretación de resultados, así como aspectos relacionados con la comunicación de resultados, y se describen otros fluidos alternativos para el análisis de ctDNA. Por último, se repasan las aplicaciones clínicas del ctDNA y se enumeran las ventajas y limitaciones que la implementación del ctDNA presenta en la práctica clínica.

## ADN circulante

### Características del ctDNA en sangre

El ADN libre circulante (cfDNA, por sus siglas en inglés) está compuesto por pequeños fragmentos de ADN de doble hélice nuclear y mitocondrial [7], principalmente liberados en la circulación sanguínea por el sistema hematológico en los individuos sanos [8]. El tamaño del cfDNA varía entre 40 y 200 pares de bases (pb), con un tamaño medio de alrededor de 166 pb, lo cual corresponde a fragmentos de ADN asociados a nucleosomas [7], 9]. En los pacientes oncológicos, el cfDNA también contiene un pequeño conjunto de fragmentos más pequeños (∼145 bp) liberados en el torrente sanguíneo por las células tumorales, llamados ctDNA [10], que son los responsables de los elevados niveles de cfDNA observados en los pacientes con cáncer [11]. El ctDNA suele estar más fragmentado que el cfDNA [12] y se caracteriza por presentar características genéticas y epigenéticas distintivas que aportan información sobre el tumor de origen [10]. La liberación de ctDNA a la circulación se puede producir a través de diversos mecanismos (Figura 1), que implican procesos de liberación tanto activos como pasivos [12], 13]. La liberación pasiva de ctDNA principalmente se produce mediante la apoptosis y la necrosis [13]. Se piensa que, en la mayoría de los tumores, la apoptosis es el principal mecanismo de liberación de ctDNA, produciendo un patrón de escalera en los fragmentos de ADN [14], con una periodicidad de 10 bp [11]. Aunque la necrosis contribuye de forma variable a la liberación de ctDNA, dicho mecanismo suele generar fragmentos de ctDNA de mayor tamaño, principalmente superiores a los 200 bp, e incluso por encima de los 10.000 bp, acompañados de un patrón característico del oligonucleosoma en escalera [13], 15], 16]. Además, el ctDNA también se puede derivar de vesículas extracelulares (esto es, exosomas, microvesículas o cuerpos apoptóticos) [17], lo cual abre la vía a nuevas líneas de investigación [18]. No obstante, su aportación exacta al ctDNA sigue siendo objeto de debate [19], 20]. Por otro lado, cabe señalar que el ctDNA tiene una vida media corta en la circulación (menos de 2 h) [21], lo que se ve influido por la degradación enzimática en el torrente sanguíneo, su depuración hepática y, en menor medida, por la acción del riñón [22]. Tradicionalmente, el método de referencia para la caracterización molecular de los tumores sólidos ha sido mediante biopsia directa de la lesión. Sin embargo, en la última década, se ha desarrollado la alternativa del análisis de ctDNA en sangre [12], existiendo evidencia creciente sobre su utilidad en la caracterización molecular no invasiva de los tumores sólidos [23], [24], [25], [26], [27]. El ctDNA ofrece la oportunidad de realizar un análisis no invasivo de los tumores, permitiendo la toma de muestras seriadas, para realizar un seguimiento de la evolución del tumor, con mejores tiempos de respuesta, un menor coste, y un proceso más simplificado, si lo comparamos con la biopsia de tejidos. Así mismo, el ctDNA permite analizar la evolución clonal o de la carga mutacional del tumor [28], pudiendo capturar la heterogeneidad de manera más eficaz que la biopsia de tejidos, que tiene una naturaleza inherentemente localizada y, en algunos tipos de cáncer, es difícil de obtener [12], 29]. De hecho, el ctDNA tiene la capacidad de capturar la heterogeneidad tumoral, alcanzando una sensibilidad de entre el 80 y el 90 % [30], dependiendo de la localización anatómica y la abundancia de ctDNA [31].

El análisis de las características de ctDNA puede resultar de utilidad en la detección de una amplia variedad de características tumorales (Figura 1) [4], 12]. La mayoría de las publicaciones se han centrado en el análisis de las variantes genéticas, como las variantes de nucleótido único (SNVs), las inserciones y deleciones *(indels),* las variaciones en el número de copias (CNVs) y las fusiones. Por otro lado, en los últimos años, otras características del ctDNA han obtenido especial atención [12]. Por ejemplo, los niveles de ctDNA están correlacionados con el tamaño tumoral, pudiendo ser empleados para evaluar la progresión de la enfermedad [7], 28], existiendo una relación entre niveles inferiores y mayor beneficio clínico [21]. Además, el análisis del perfil del tamaño de los fragmentos de cfDNA y ctDNA en los pacientes oncológicos puede aportar información potencialmente valiosa sobre el tejido de origen [32], con posibles aplicaciones para mejorar la detección de ctDNA [33]. No menos importante es el perfil de metilación del ctDNA, que también puede proporcionar información importante sobre el tumor [5], 34], tal como el tejido de origen [12], 35].

### El ctDNA en fluidos corporales no sanguíneos

Aparte de en la sangre, se puede encontrar ctDNA en otros fluidos biológicos, con una mayor sensibilidad en determinados contextos [36], 37], presentando cada uno diferentes ventajas e inconvenientes [7], 37]. Sin embargo, su aplicación en los análisis clínicos rutinarios se ve limitada por la falta de estandarización.

La orina es una prometedora fuente no invasiva de ctDNA para la detección de tumores genitourinarios [38], [39], [40], [41], dado que puede mejorar la detección y el manejo de estos tipos de tumores [42], [43], [44]. Otra fuente no invasiva de ctDNA es la saliva, que permite la toma de muestras seriadas para el análisis de los tumores de cabeza y cuello [45], [46], [47], [48]. Algunos estudios señalan al ctDNA de la saliva como el tipo de muestra más adecuado para los tumores orales, pudiendo emplearse en combinación con ctDNA extraído de la sangre para otros tumores de cabeza y cuello [37], 49]. Por otro lado, el líquido cefalorraquídeo (LCR) es especialmente útil para el diagnóstico y el seguimiento de los tumores primarios del sistema nervioso central y las metástasis cerebrales [50], [51], [52], [53], [54]. Dado que la barrera hematoencefálica limita en gran medida el paso de ctDNA del torrente sanguíneo al sistema nervioso central [55], el líquido cefalorraquídeo es una fuente potencial específica de ctDNA cerebral [37]. Esta estrategia elimina la necesidad de realizar una biopsia del tumor cerebral, permitiendo además la toma de muestras en serie [56]. Además de estos fluidos, el análisis de ctDNA en derrames también presenta algunas ventajas, dado que dichos fluidos poseen una mejor relación ctDNA-cfDNA que la sangre para los tumores proximales [57]. Por ejemplo, el ctDNA en líquido pleural permite detectar rápidamente las mutaciones accionables con terapia dirigida *(actionable mutations)* en el cáncer de pulmón, con una sensibilidad superior a la del ctDNA obtenido en sangre [37]. Así mismo, se ha demostrado la utilidad del ctDNA en líquido peritoneal para la detección de enfermedad peritoneal metastásica [37], 58]. Otros tipos de fluidos, como los lavados bronquiales, ofrecen la posibilidad de analizar el ctDNA, con un valioso potencial en el diagnóstico del cáncer de pulmón [59]. Este tipo de fluido contiene mayores niveles de ctDNA que la sangre, resultando especialmente útil para la caracterización molecular de los tumores pulmonares [59], [60], [61]. Además, se están investigando otras posibles fuentes de ctDNA con potencial aplicación clínica, como la bilis en el cáncer de páncreas [62], y la leche materna de mujeres embarazadas y en periodo de lactancia para el cáncer de mama [63], entre otras posibles fuentes.

## Aspectos preanalíticos en el análisis de ctDNA

Los aspectos preanalíticos (Tabla 1) son esenciales a la hora de garantizar la fiabilidad del análisis del ctDNA, ya que un manejo o procesamiento inadecuado puede provocar la contaminación de la muestra, así como su degradación, o derivar en un bajo rendimiento [4], 64].

**Tabla 1:** Consideraciones preanalíticas para el análisis de ctDNA.

Característica	Consideraciones
Tipo de muestra	Se recomienda el plasma frente al suero.
Extracción de sangre	Los tubos EDTA deben ser procesados en las siguientes 2–4 horas. Los tubos con conservantes celulares mantienen la integridad de la muestra durante varios días a temperatura ambiente.
Transporte de sangre	Se debe evitar agitar la muestra, así como las fluctuaciones de temperatura. Los tubos EDTA deben llegar al laboratorio en 2–4 horas. Los tubos con conservantes celulares se pueden transportar a temperatura ambiente durante varios días sin que se produzca una degradación significativa. Para tiempos de transporte más prolongados, se debe separar y congelar el plasma.
Separación del plasma y QC	Normalmente consta de dos pasos de centrifugación. Se debe obtener el plasma sin alterar la capa leucocitaria (buffy coat) ni los glóbulos rojos. Se debe evitar la hemólisis, la lipemia y la ictericia.
Condiciones de conservación de plasma	Para un almacenamiento prolongado (meses): −80 °C. Para un almacenamiento breve (hasta 30 días): −20 °C. Evitar la repetición de ciclos de congelado-descongelado que puedan provocar la fragmentación del ctDNA y la pérdidad de sensibildiad analítica.
Métodos de extracción	Se pueden emplear métodos de extracción manual o automatizada. El rendimiento y la pureza son relevantes a la hora de seleccionar la metodología.
QC y almacenamiento del ctDNA	La fluorométría o PCR cuantitativa se suelen emplear para determinar la concentración de ctDNA. El ctDNA se suele almacenar a −80 °C si no se va a utilizar de forma inmediata, debiendo evitar repetir ciclos de congelado-descongelado.

QC, control de calidad.

### Tipos de muestra

Para el análisis de ctDNA, es preferible emplear plasma que suero. Durante el proceso de coagulación en suero, los leucocitos pueden liberar ADN genómico (gDNA), incrementando así la contaminación de la muestra, y dificultando la detección de mutaciones de baja frecuencia. El plasma minimiza la contaminación de gDNA, proporcionando unos resultados más fiables en la detección de alteraciones de baja frecuencia [65].

### Recogida y transporte de muestras sanguíneas

Dependiendo del uso que se vaya a hacer de los resultados del análisis de ctDNA, seleccionar correctamente el momento en el que realizar la extracción de sangre resulta esencial. Previamente al tratamiento, el análisis de ctDNA permite establecer los valores iniciales de ctDNA y carga tumoral. Durante el tratamiento, permite realizar un seguimiento de la eficacia terapéutica y detectar posibles resistencias. Finalmente, el análisis de ctDNA tras el tratamiento permite la identificación precoz de recurrencias y de progresión de la enfermedad [5], 21], 66]. Para la extracción de sangre, se pueden emplear tubos con o sin conservantes [67]. Los tubos sin conservantes más comunes en el análisis de ctDNA son aquellos que contienen el anticoagulante etilendiaminotetraacetato de potasio (K_2_EDTA). Cabe señalar que, cuando se obtienen muestras de sangre en tubos K_2_EDTA, es necesario procesarla previamente entre 2 y 4 h después de la extracción para prevenir la lisis celular y la contaminación por gDNA. Por otro lado, los tubos con conservantes (por ejemplo, los tubos Streck) están diseñados específicamente para estabilizar las células sanguíneas nucleadas, permitiendo la conservación del ctDNA en muestras de sangre almacenadas durante varios días a temperatura ambiente [1], 68], 69]. Los tubos sanguíneos deben ser transportados al laboratorio sin agitarse y protegidos de las oscilaciones térmicas, para evitar la hemólisis y el daño celular [70]. Si se trabaja con laboratorios externos, es importante plantearse emplear tubos de conservación celular y ajustarse a los tiempos y temperatura de almacenamiento adecuados [64].

### Separación de plasma y control de calidad

El proceso de separación de plasma suele consistir en dos pasos: ∼1.600 × *g* a 4 °C durante 10 min, y una segunda centrifugación a ∼16.000 × *g* a 4 °C durante 10 min para obtener plasma libre de células [5]. Este proceso ayuda a eliminar desechos celulares, mejorando la pureza del ctDNA [65]. Factores como la hemólisis, la lipemia y la ictericia pueden interferir en el análisis del ctDNA, por lo que se recomienda realizar una inspección visual del plasma. Para minimizar la hemólisis, se aconseja realizar una venopunción suave e invertir inmediatamente los tubos de almacenamiento [4], 64]. Se recomienda desechar las muestras hemolizadas [67], 70], dado que en estas muestras se puede favorecer la liberación de gDNA, interfiriendo así en el proceso de extracción y reduciendo la proporción de ctDNA [67], 71]. Con respecto a la lipemia y la ictericia, es necesario realizar más estudios para determinar el efecto de los niveles elevados de bilirrubina o el impacto de la hiperlipidemia en los niveles de ctDNA.

### Condiciones de almacenamiento del plasma

El plasma se puede congelar a −20 °C durante un máximo de 30 días, si se prevé realizar el análisis a corto plazo [1], 65]. Para un almacenamiento más prolongado, se debe congelar a −80 °C. La preparación adecuada de alícuotas es esencial a la hora de evitar la repetición de ciclos de congelado y descongelado, que pueden provocar la fragmentación del ADN y reducir la cantidad de ctDNA, afectando así a la precisión de su análisis [72].

### Métodos de extracción de ctDNA

Los métodos de extracción se deben adaptar a las características del ctDNA, que se suele encontrar a concentraciones bajas y en fragmentos de pequeño tamaño. Actualmente, existen en el mercado varios kits de extracción de ctDNA, lo que permite una adecuada recuperación y reproducibilidad [65]. Los laboratorios deben seleccionar el método más adecuado, teniendo en cuenta tanto el rendimiento como la pureza del ADN de bajo peso molecular. Se pueden emplear tanto procedimientos manuales como automáticos, dependiendo del rendimiento de la plataforma y de las necesidades específicas del laboratorio [64].

### Evaluación de la calidad y almacenamiento de ctDNA

La evaluación de la calidad del ctDNA resulta esencial en el análisis de los eventos moleculares. La cuantificación por fluorometría se suele emplear para medir la concentración de ctDNA, aunque los métodos de electroforesis resultan de utilidad a la hora de verificar el tamaño de los fragmentos de cfDNA y confirmar la ausencia de contaminación del gDNA. Cuando no está previsto analizar el ctDNA de manera inmediata, este se debe congelar a −80 °C en múltiples alícuotas, para evitar la degradación que se produce en los ciclos de congelado y descongelado [72], 73].

## Aspectos analíticos en el análisis de ctDNA

Al analizar el ctDNA, se deben tener en cuenta una serie de factores analíticos (Tabla 2) que suelen interferir en los resultados de las variantes somáticas, como la hematopoyesis clonal y la presencia de variantes germinales. Del mismo modo, se debe plantear el uso de controles de calidad internos y externos, así como realizar una minuciosa validación analítica, para optimizar la detección de ctDNA en la práctica habitual, garantizando la fiabilidad y la aplicabilidad clínica de las pruebas.

**Tabla 2:** Consideraciones analíticas sobre las pruebas de ctDNA.

Característica	Consideraciones
Hematopoyesis clonal (CHIP)	La existencia de CHIP dificulta la interpretación de ctDNA al poder generar falsos positivos. La secuenciación de PBMCs evita resultados confusos debidos a la presencia de CHIP.
QC interno y externo	El QC interno y externo ayuda a evaluar la calidad del análisis de ctDNA.
Variantes germinales	Las variantes germinales patogénicas en los genes de predisposición al cáncer se pueden detector mediante el análisis de ctDNA.
Validación analítica	La sensibilidad analítica (límite de detección) y la precision analítica son parámetros de calidad analítica esenciales en la evaluación de las pruebas de ctDNA.
Limitaciones analíticas	Sensibilidad limitada con respecto al genotipado de tejidos debido a: i) VAF baja y elevada fragmentación del ctDNA, ii) heterogeneidad clonal, caracterizada por multiples clones tumorales con diferentes mutaciones a una VAF baja, iii) niveles elevados de cfDNA que diluyen el ctDNA, y iii) diseminación limitada en los tumores en etapa temprana y baja carga tumoral.

QC, control de calidad; PBMCs, células mononucleares de sangre periférica.

### Hematopoyesis clonal

La hematopoyesis clonal de potencial indeterminado (CHIP, por sus siglas en inglés) es un proceso asociado a la edad, a través del cual las mutaciones somáticas de las células madre hematopoyéticas provocan la expansión clonal [4], 74]. Dado que el cfDNA se deriva en gran medida de las células hematopoyéticas, la existencia de CHIP dificulta la interpretación del ctDNA, pudiendo generar falsos positivos, especialmente en los genes asociados a los tumores sólidos, como los genes *KRAS*, *GNAS*, *NRAS*, y *PIK3CA* [75]. Para la interpretación precisa de variantes, es importante diferenciar adecuadamente las mutaciones relacionadas con CHIP de aquellas derivadas del tumor, lo que suele implicar la secuenciación tanto del cfDNA como de células mononucleares de sangre periférica (PBMC) [76]. Las estrategias basadas en el análisis del tamaño de los fragmentos de cfDNA también pueden aumentar la precisión de la interpretación de las variantes [33], 77].

### Variantes germinales

A la hora de evaluar los resultados de ctDNA, se debe tener en cuenta la posible detección accidental de variantes germinales patogénicas (PGVs), especialmente en las pruebas de secuenciación de nueva generación (NGS) que incluyen genes de predisposición a cáncer (como *BRCA1*, *BRCA2*, *PALB2*) [4], 78], 79]. A este respecto, la presencia de una frecuencia alélica de variante (VAF, por sus siglas en inglés) en circulación de entre el 40 y el 60 % es indicativa de un origen germinal, ya que las variantes somáticas suelen presentar VAF más bajas [80]. Sin embargo, el ctDNA puede mostrar niveles elevados de VAF, debido a la presencia de una elevada carga tumoral o la pérdida de heterocigosidad [78], 81]. Únicamente las variantes clasificadas como “patogénicas” y “probablemente patogénicas” según la guía del American College of Medical Genetics and Genomics [82], ClinVar, y demás fuentes, se deben tener en cuenta como posibles variantes germinales patogénicas [79]. Según las recomendaciones de la Sociedad Europea de Oncología Médica (ESMO), cuando se sospecha la presencia de una posible variante germinal, se debe realizar un análisis genético de línea germinal mediante una prueba validada, con el fin de confirmar su origen germinal o somático [4]. De este modo, resulta esencial avisar al clínico de la posible detección de mutaciones germinales o relacionadas con CHIP, especialmente en las pruebas de ctDNA dirigidas a los genes que suelen presentar mutaciones.

### QC externo e interno

La implementación de controles de calidad (QC) internos y externos resulta crucial a la hora de garantizar la veracidad y reproducibilidad en la detección de variantes genéticas en el ctDNA de pacientes oncológicos. Existen controles internos comerciales, como el *Structural Multiplex cfDNA Reference Standard* (HD786) de Horizon Discovery, para evaluar la calidad del análisis de ctDNA [83]. Por otro lado, las evaluaciones externas de calidad (EQA), como las que ofrece la Red Europea de Calidad Genética Molecular (EMQN), ofrecen programas de evaluación de calidad para la detección de variantes de ctDNA (p.ej., “LUNG CANCER (NSCLC) [Plasma],” “cfDNA Multiple Biomarkers”). El empleo de dichos controles puede ayudar a detectar posibles errores técnicos, garantizando así la fiabilidad de los resultados clínicos, que son esenciales para la toma de decisiones terapéuticas basadas en el uso de biomarcadores. La validación continua a través de calibradores internos y la comparación con programas externos de calidad garantiza la solidez de los análisis de ctDNA [83], [84], [85].

### Validación analítica

Se debe realizar una validación analítica para optimizar la detección de ctDNA en la práctica clínica habitual, debiendo adaptarla a la población específica de pacientes y a la indicación médica de la prueba. Las recomendaciones y protocolos para la validación de las pruebas de ctDNA incluyen evaluar los parámetros de calidad analítica, como la sensibilidad analítica, la veracidad, la repetibilidad, la precisión y la reproducibilidad [86], 87]. Los laboratorios deben establecer y evaluar el límite de detección de cada clase de variante para garantizar la fiabilidad de los resultados para variantes a bajas frecuencias [86]. La veracidad analítica se puede evaluar mediante la comparación de métodos (comparando los resultados con los obtenidos con un método ortogonal) o utilizando materiales de referencia conocidos [87]. Entre las pruebas ortogonales de confirmación para la validación analítica se encuentran la PCR cuantitativa, la PCR digital (dPCR), la PCR digital por gotas (ddPCR), la secuenciación de ADN, o cualquier método con una sensibilidad igual o superior a la de la prueba que se pretende validar [86].

### Limitaciones analíticas

La principal limitación del análisis de ctDNA es su menor sensibilidad frente al genotipado de tejidos, lo que deriva en una mayor tasa de falsos negativos. Esta menor sensibilidad se debe a diferentes factores, entre los que se incluyen una concentración extremadamente baja, una fragmentación elevada del cfDNA en plasma, así como la baja proporción de ctDNA en el conjunto total de cfDNA, que suele oscilar entre 0,01 % y 0,1 %. Además, la heterogeneidad clonal y la elevada concentración de cfDNA normal, que a menudo proviene de patologías no malignas o de inflamación postoperatoria, diluyen aún más el ctDNA, dificultando más si cabe la detección de variantes de baja frecuencia [88], 89]. La fiabilidad de la detección se ve especialmente afectada por las mutaciones con VAF bajas. Otros factores son un estadio temprano del tumor, una baja carga tumoral, y los tumores no diseminados, que, en su conjunto, pueden reducir las tasas de detección. La cantidad de cfDNA constituye así mismo una variable esencial, ya que una mayor cantidad se asocia a una mayor profundidad de lectura de los fragmentos y a una mejor sensibilidad y reproducibilidad [90]. Por otro lado, la detección de falsos positivos en el análisis de ctDNA es relativamente poco frecuente. Aunque los falsos positivos se suelen producir en las variantes de baja frecuencia, estos se pueden minimizar aplicando identificadores moleculares únicos (UMI, por sus siglas en inglés) y estableciendo un valor límite mínimo de VAF, normalmente superior al 0.05 %, lo que reduciría el impacto de los errores de secuenciación [90].

## Métodos para el análisis de variantes genéticas en ctDNA

En los últimos años, tanto la comunidad científica como la industria del diagnóstico *in vitro* han desarrollado múltiples aplicaciones para el análisis de ctDNA en tumores sólidos (Figura 1). Sin embargo, debido a la complejidad y las limitaciones de estos análisis moleculares, su uso se ha visto restringido a la investigación clínica, habiendo sido aprobados únicamente unos pocos para el diagnóstico *in vitro*. Aunque diferentes pruebas ya han recibido aprobación por parte de las autoridades sanitarias (p.ej. Guardant360 CDx, FoundationOne Liquid CDx) para distintas aplicaciones clínicas [91], esta revisión se va a centrar en las pruebas y tecnologías que se podrían integrar en los laboratorios clínicos para su uso en la práctica habitual. En la Tabla 3 se muestran los métodos comerciales más habituales, así como la tecnología o el equipamiento que estos utilizan, su estado de regulación, los marcadores moleculares, las especificaciones técnicas de las pruebas, los tiempos de respuesta y sus aplicaciones.

**Tabla 3:** Pruebas de ctDNA más habituales disponibles en el mercado para los laboratorios clínicos.

Prueba–Compañía	Tecnología	Equipamiento	Estado de regulación	Marcadores moleculares	Principales especificaciones del estudio^a^	Aplicación en la clínica y en investigación^a^
Prueba de mutaciones Cobas^®^ EGFR v2 – Roche	RT- PCR	Analizador Cobas z 480	CE-IVD/US-IVD	Mutaciones/Indels * EGFR*	LOD: Menos de 100 copias de ADN mutado por mL de plasma Tiempo de respuesta: 4 horas desde la extracción de sangre hasta la emisión de resultados	Cáncer de pulmón
Pruebas Idylla Mutation – Biocartis	RT- PCR	Programa Biocartis Idylla™	RUO	Mutaciones * KRAS* * NRAS/BRAF* * EGFR*	LOD < 5 % para todas las mutaciones *KRAS* y las mutaciones *EGFR* más prevalentes Tiempo de respuesta: 3 horas desde la extracción de ADN libre de células hasta la obtención de resultados	Cáncer de pulmón y colorrectal
Kits de PCR Therascreen – Qiagen	RT- PCR	Rotor-Gene^®^ Q MDx 5plex HRM	CE-IVD/US-IVD/RUO^b^	Mutaciones * PIK3CA* * EGFR*	Porcentaje general de concordancia plasma-tejido 72 % Tiempo de respuesta: 1–2 días desde la extracción de la sangre hasta la comunicación de los resultados	Cáncer de pulmón y de mama
Kits de Plasma-SeqSensei™ – Sysmex	Multiplex PCR–NGS	Plataformas de secuenciación Illumina NextSeq 500/550 y MiSeq	CE-IVD /RUO^b^	Mutaciones/Indels Kit para tumores sólidos (*BRAF*, *EGFR*, *KRAS*, *NRAS* y *PIK3CA*) Kit para cáncer de mama (*AKT1*, *ERBB2*, *ESR1*, *KRAS*, *PIK3CA* y T*P53*) NSCLC Kit (*EGFR*, *KRAS*, *BRAF* y *PIK3CA*) Kit para cáncer colorrectal (*KRAS*, *NRAS*, *BRAF* y *PIK3CA*)	LOD: 0,06 % MAF. Tiempo de respuesta: 2 días desde la extracción de ADN libre de células hasta la obtención de resultados, incluyendo el proceso de secuenciación	Tumores sólidos, cáncer de pulmón, colorrectal y de mama
Paneles de NGS de Oncomine – ThermoFisher	Librerías basadas en amplicones para NGS	Sistema Ion GeneStudio S5 y sistema Ion Torrent Genexus	RUO	Mutaciones/fusiones/indels/CNVs Estudio panoncológico basado en ADN circulante (52 genes) Estudio de precisión (50 genes) Estudio de cfTNA para cáncer de pulmón (12 genes) Estudio de cfDNA para cáncer de mama (12 genes) Estudio de cfDNA para el cáncer de colon (14 genes)	LOD: hasta 0,1 % VAF para las regiones críticas con acumulación de SNV e indels Tiempo de respuesta: 1–3 días desde la extracción de sangre hasta la comunicación de los resultados	Pan-cáncer, cáncer de pulmón, de mama y colorrectal
Paneles de NGS de Avenio – Roche	Librerías basadas en captura híbrida para NGS	Plataforma de secuenciación Illumina NextSeq 500/550	RUO	Mutaciones/fusiones/indels/CNVs Kit dirigido (17 genes) Kit ampliado (77 genes) Kit de vigilancia (197 genes)	LOD: hasta 0,5 % VAF Tiempo de respuesta: 5 días desde la extracción de cfDNA hasta la comunicación de resultados	Pan-cáncer
TruSight Oncology 500 ctDNA – Illumina	Librerías basadas en captura híbrida para NGS	Plataforma de secuenciación Illumina NovaSeq X/NovaSeq 6,000	RUO	Mutaciones/fusiones/indels/CNVs/MSI/TMB TruSight Oncology 500 ctDNA (523 genes)	LOD para variantes pequeñas: 0.5 % VAF Tiempo de respuesta: 3–4 días desde la obtención de ácido nucleico purificado hasta la comunicación de la presencia de variantes	Pan-cáncer
Guardant360 CDx – Guardant Healthde	Librerías basadas en captura híbrida para NGS	Aplicación comercial subcontratada	Aprobado por la FDA	Mutaciones/indels (74 genes)/fusiones (6 genes)/amplificaciones (18 genes)/MSI	LOD para las SNV varía según la cantidad de cfDNA 0,2 % de VAF a 30 ng y 1,8 % de VAF a 5 ng. Tiempo de respuesta: 7 días desde la recepción de la muestra hasta la obtención de los resultados	Tumores sólidos. Prueba utilizada para identificar pacientes elegibles para terapias dirigidas
FoundationONE Liquid CDx – Foundation Medicine	Librerías basadas en captura híbrida para NGS	Aplicación comercial subcontratada	Aprobado por la FDA	Mutaciones/indels (311 genes)/reordenamientos (4 genes)/amplificaciones (3 genes)/MSI/TMB	La mediana del LOD para las variantes cortas oscila entre el 0,4 % y el 0,8 % de la VAF, según la región genómica. Tiempo de respuesta: 8 días desde la recepción de la muestra hasta la obtención de los resultados	Tumores sólidos. Prueba utilizada para identificar pacientes elegibles para terapias dirigidas
Signatera – Natera	Secuenciación completa del exoma + secuenciación múltiple de nueva generación basada en PCR	Aplicación comercial subcontratada	Designación de dispositivo innovador	Ensayo personalizado, basado en perfil de mutaciones único del tumor de cada paciente	LOD: 0,01 % VAF Tiempo de respuesta: 3 semanas para la secuenciación tumoral inicial y el diseño del ensayo personalizado; 1-2 semanas para los resultados de MRD desde la recepción de la muestra	Multicáncer. Prueba utilizada para el seguimiento del tratamiento y la evaluación de la enfermedad mínima residual (MRD)

^a^Según las especificaciones del fabricante. ^b^El estado de regulación depende de la prueba específica. IVD, diagnóstico *in vitro*; RUO, solo para fines de investigación; LOD, límite de detección; RT-PCR, PCR en tiempo real; NGS, secuenciación de nueva generación.

Entre las tecnologías moleculares actuales para el análisis de ctDNA se incluyen los métodos basados en PCR y las tecnologías basadas en NGS. Las técnicas basadas en PCR están diseñadas para identificar alteraciones genéticas concretas, e incluyen la PCR cuantitativa en tiempo real (qPCR) y la dPCR. La principal ventaja de estas técnicas frente a los paneles de secuenciación basados en NGS radica en su elevada sensibilidad y especifidad para la detección de variantes, con capacidad para identificar VAFs al 0,1 % o porcentajes inferiores [92]. Sin embargo, los métodos basados en PCR únicamente pueden detectar un número limitado de variantes conocidas, mientras que la NGS facilita la identificación simultánea de múltiples marcadores en varias muestras de una misma carrera.

Actualmente, existen varios productos comerciales basados en qPCR. Las pruebas de qPCR en tiempo real son más sencillas y están diseñadas para aplicaciones clínicas concretas, algunas de las cuales han sido aprobadas para su uso en la rutina clínica. Las pruebas basadas en dPCR incluyen numerosas pruebas desarrolladas para el sistema BioRad QX200/QX600 Droplet Digital PCR y el sistema de dPCR Thermo Fisher Scientific Absolute Q, ambos con sensibilidades similares [93]. Sin embargo, tanto los reactivos como el equipamiento únicamente están disponibles en la versión RUO (del inglés, Research Use Only), por lo que estas pruebas solo están disponibles para investigación clínica. Para que estas pruebas se puedan emplear en la clínica, resulta esencial realizar una validación analítica y clínica rigurosa.

Los métodos de análisis de ctDNA basados en NGS permiten la detección de alteraciones en un amplio espectro de genes. Entre los paneles de NGS disponibles actualmente en el mercado para el análisis de ctDNA se encuentran las pruebas Oncomine NGS (Thermo Fisher); los kits Avenio ctDNA (Roche); TruSight Oncology 500 ctDNA (Illumina); y los paneles QIAseq Targeted cfDNA Ultra (Qiagen), entre otras. Estos paneles difieren en los genes o regiones que son cubiertas, los tipos de alteraciones que pueden detectar, y en su sensibilidad para la detección de variantes. Sin embargo, estas pruebas suelen detectar mutaciones de ctDNA con una frecuencia superior al 0.5 % con una elevada sensibilidad, precisión y reproducibilidad [90].

En la práctica clínica, dada la amplia variedad de pruebas actualmente existentes para el análisis de ctDNA, a la hora de seleccionar la prueba más adecuada, se debe tener en cuenta su disponibilidad, financiación, y el número de alteraciones accionables en el contexto de un tipo específico de tumor [4].

## Interpretación completa de los resultados de variantes genéticas en las pruebas de ctDNA

Las recomendaciones para identificar, interpretar e informar de la presencia de variantes en el análisis de cfDNA se deben ajustar a los criterios establecidos para la interpretación de variantes somáticas y la clasificación de oncogenicidad [94]. No obstante, es necesario tener en cuenta las características únicas del ctDNA y ajustarse a las guías específicas adaptadas al análisis de ctDNA en los distintos tipos de tumores [4], 95].

La identificación de variantes en el análisis de ctDNA implica la detección de SNVs, *indels,* fusiones y CNVs. Aunque muchas herramientas de software automatizan este proceso, los laboratorios clínicos deben ser conscientes de sus limitaciones, dado que el análisis de ctDNA presenta algunas dificultades a la hora de identificar con precisión ciertas aberraciones genéticas, como las CNVs o las variantes de fusión [4], 96]. Para una interpretación precisa, es necesario evaluar meticulosamente métricas esenciales, como la profundidad de secuenciación (cobertura) y las VAFs [97]. A la hora de interpretar los hallazgos en el ctDNA, es necesario tener en cuenta que estas pruebas tienen una menor sensibilidad que la caracterización molecular de tejido, lo que puede incrementar la probabilidad de falsos negativos. Así mismo, en el análisis de ctDNA, es importante considerar la posibilidad de que se produzcan falsos positivos debido a la identificación de variantes CHIP, que se pueden detectar a bajas VAFs (0.1–5 %), lo que puede llevar a su interpretación errónea como variantes derivadas del tumor [98]. Dado que algunas patologías benignas esporádicas pueden presentar alteraciones somáticas en genes impulsores del cáncer, los resultados de los ensayos de ctDNA deben interpretarse en el contexto clínico del paciente. Por ejemplo, la variante V600E en ADN plasmático no solo se ha encontrado en pacientes con cáncer, sino también en individuos con nevus benigno [99].

Se recomienda clasificar las variantes genéticas según su accionabilidad, aplicando la evidencia científica disponible en el diagnóstico, pronóstico y la elegibilidad para terapias aprobadas por la FDA o la EMA, o ensayos clínicos. En este sentido, el sistema de clasificación por niveles *(Tier system)* de la Asociación de Patología Molecular (AMP) y la Escala ESMO para la Accionabilidad Clínica de las Dianas Moleculares (ESCAT) evalúan las alteraciones genéticas en función de su relevancia clínica. La clasificación por niveles de la AMP categoriza las variantes somáticas en cuatro niveles, en función de su significación clínica de la siguiente manera: el Tier I incluye las variantes de gran relevancia clínica. El Tier II denota variantes con posible relevancia clínica. El Tier III hace referencia a las variantes de significado clínico desconocido, mientras que el Tier IV se compone de variantes consideradas benignas o probablemente benignas. Los niveles de evidencia A o B (Tier I) y C o D (Tier II) se ponderan según su relevancia en la toma de decisiones clínicas [97]. Por otro lado, la escala ESCAT clasifica las aberraciones moleculares del Tier I al Tier V y X, fundamentándose en la evidencia disponible sobre su valor como dianas terapéuticas. En el Tier I se incluyen las alteraciones moleculares para las que existe un fármaco específico recomendado para uso habitual, mientras que para el resto de niveles de evidencia clínica (ESCAT Tier II a V) no se dispone de suficiente información, por lo que su aplicación se limita al campo de los ensayos clínicos. Para las alteraciones de ESCAT Tier X, no existe evidencia clínica o preclínica, por lo que estas no deben ser tenidas en cuenta en las decisiones terapéuticas [100].

Bases de datos como COSMIC, ClinVar, y OncoKB son herramientas esenciales para la interpretación de los resultados del análisis de ctDNA de los pacientes oncológicos (Tabla 4). Estas plataformas aportan contexto a las variantes genéticas detectadas, al recoger datos sobre variantes somáticas, su patogenicidad y relevancia clínica en los diferentes tipos de cáncer, facilitando la identificación de variantes de significado incierto (VUS).

**Tabla 4:** Bases de datos habitualmente empleadas para la interpretación de variantes genéticas relacionadas con el cáncer.

Base de datos	Descripción	URL
Cancer Genome Interpreter (CGI)	Incluye alteraciones tumorales impulsoras de la enfermedad y que pueden ser tratables mediante terapia dirigida. Emplea métodos computacionales como la mutagénesis por saturación *in silico* de genes relacionados con el cáncer (BoostDM and OncodriveMut) [109].	https://www.cancergenomeinterpreter.org
Cancer Hotspots	Incluye mutaciones recurrentes significativas identificadas en datos genómicos del cáncer a gran escala, detectadas en muestras tumorales mediante el algoritmo descrito [110].	https://www.cancerhotspots.org
cBioPortal	Plataforma interactiva y de código abierto diseñada para la visualización, exploración y análisis de datos genómicos del cáncer y de variantes somáticas en los distintos tipos de tumores [111].	https://www.cbioportal.org
CiVIC (Clinical Interpretation of Variants in Cancer)	Proporciona interpretaciones clínicamente relevantes de las variantes genéticas del cáncer para respaldar la toma de decisiones terapéuticas, y facilita la colaboración entre investigadores, clínicos y representantes de los pacientes [112].	https://civicdb.org
CKB Core (Jackson Laboratory Clinical Knowledgebase)	Recurso digital dinámico para interpretar perfiles genómicos complejos del cáncer en términos de impacto proteico, terapéutico y de ensayos clínicos [113].	https://ckb.jax.org
ClinVar	Archivo público que cataloga las variantes genéticas humanas asociadas a enfermedades, respuestas a tratamientos y patologías malignas, mejorando la comunicación y facilitando la reevaluación de la clasificación de variantes [114].	https://www.ncbi.nlm.nih.gov/clinvar
COSMIC (Catalogue of Somatic Mutations in Cancer)	Fuente de información sobre mutaciones somáticas revisadas por expertos relacionadas con enfermedades oncológicas humanas, que ofrece un completo catálogo de variantes somáticas y de genes asociados con el cáncer [115].	https://cancer.sanger.ac.uk/cosmic
DoCM (Database of Curated Mutations)	Repositorio curado que combina información sobre genes y variantes con valor pronóstico, diagnóstico, predictivo o funcional, a partir de diferentes fuentes y publicaciones individuales [116].	https://docm.info
Franklin	Plataforma impulsada por inteligencia artificial que automatiza el flujo de trabajo desde datos de secuenciación crudos (FASTQ/VCF, por sus siglas en inglés) hasta la generación de informes clínicos, ofreciendo un análisis integral de variantes, evidencia bibliográfica, clasificación automatizada basada en los criterios ACMG, así como herramientas de anotación y evaluación [117].	https://franklin.genoox.com
My Cancer Genome	Proporciona información sobre el impacto clínico de los biomarcadores moleculares en el uso de fármacos en oncología, basada en las fichas técnicas de la FDA, las guías de la NCCN, ensayos clínicos y publicaciones revisadas por pares, empleando datos de las muestras tumorales en la base de datos GENIE del proyecto AACR [118].	https://www.mycancergenome.org
PMKB (Precision Medicine Knowledgebase)	Interfaz para la edición colaborativa y el intercambio de interpretaciones de mutaciones cancerígenas de calidad clínica, diseñada para facilitar la recopilación, el mantenimiento y la generación de informes interpretativos, en el contexto de pruebas genómicas oncológicas de uso clínico [119].	https://pmkb.weill.cornell.edu
OncoKB	Centrada en la oncología de precisión, aportando datos biológicos y clínicos sobre alteraciones genómicas en el cáncer. Las alteraciones y las implicaciones terapéuticas específicas de cada tipo de tumor se clasifican según los niveles de evidencia OncoKB™ [120].	https://www.oncokb.org
VarSome Clinical	Plataforma para el descubrimiento y anotación de variantes y la interpretación de datos NGS, que integra bases de datos y algoritmos públicos para aportar información detallada sobre patogenicidad de variantes, frecuencia poblacional y significación clínica [121].	https://clinical.varsome.com/

AACR, American Association for Cancer Research (Asociación Americana para la Investigación del Cáncer); ACMG, American College of Medical Genetics and Genomics (Colegio Americano de Genética Médica y Genómica); FDA, Food and Drug Administration (Administración de Alimentos y Medicamentos); NCCN, National Comprehensive Cancer Network (Red Nacional Integral del Cáncer); NGS, secuenciación de nueva generación.

La creación de comités moleculares oncológicos compuestos por un equipo multidisciplinar de profesionales sanitarios resulta esencial para poder interpretar con precisión las variantes genéticas detectadas en las pruebas de ctDNA. En estos equipos multidisciplinares, los profesionales de laboratorio desempeñan una labor crucial a la hora de evaluar la relevancia clínica de los hallazgos moleculares, ya que estos garantizan que las alteraciones genéticas sean interpretadas con exactitud y en el contexto clínico general del paciente. Los comités moleculares ofrecen una perspectiva crítica, especialmente en los casos complejos sobre los que existen datos inciertos o contradictorios. Estos esfuerzos de cooperación mejoran la calidad asistencial, al integrar diferentes perspectivas y campos de especialidad, lo que deriva en mejores resultados clínicos [101].

## Aplicaciones clínicas del ctDNA

### Aplicaciones actualmente recomendadas: enfermedad avanzada

En la práctica clínica, las pruebas de ctDNA se consideran fiables para el genotipado del cáncer avanzado y la selección de terapias moleculares dirigidas, especialmente en situaciones donde no se dispone de biopsias de tejido óptimas, o donde el tiempo sea un factor crucial [4]. La utilidad clínica de estas pruebas para orientar la terapia en presencia de variantes accionables Tier I, está respaldada por estudios prospectivos recientes de gran escala centrados en el ctDNA. Estos estudios han demostrado una elevada precisión en la detección de SNVs (en estudios comparativos entre tejido y plasma) en diversos tipos de cáncer [27], 102], 103].

Las pruebas de ctDNA han mostrado una elevada sensibilidad para la detección de SNVs y de *indels* de pequeño tamaño. Sin embargo, estas pruebas pueden mostrar una sensibilidad reducida para las fusiones, las CNVs o la inestabilidad de microsatélites (MSI), por lo que únicamente deben emplearse cuando no sea viable realizar un análisis de tejidos [4], 104]. En este contexto, un resultado negativo para la presencia de alteraciones genéticas accionables con terapias dirigidas se debe considerar no informativa si no existe evidencia de la presencia de niveles suficientes de ctDNA en la prueba. En estos casos, se recomienda confirmar el resultado mediante un análisis del tejido [4], 105]. Aunque la carga mutacional del tumor (TMB) ha demostrado poseer potencial como biomarcador predictivo en la inmunoterapia, esta sigue siendo objeto de investigación [4], 106].

Actualmente, las recomendaciones generales para el empleo de ctDNA en diferentes tipos de tumores se centran principalmente en pacientes que no disponen de resultados de pruebas genómicas en tejido cuando dichas pruebas estén indicadas, no se dispone de tejido tumoral almacenado, o no es factible realizar nuevas biopsias del tumor [4]. En la Tabla 5 se presentan las recomendaciones concretas de la ESMO para el uso de las pruebas de ctDNA en la práctica clínica habitual, incluyendo los marcadores moleculares accionables Tier I (escala ESCAT) y los fármacos asociados aprobados por la FDA.

**Tabla 5:** Aplicaciones del ctDNA en la detección de variantes Tier I según ESCAT en el manejo del cáncer en estadios avanzados.

Tipo de tumor	Gen	Aberraciones	Fármacos/Terapia^a^	Recomendación ESMO para el análisis de ctDNA [4]
Cáncer de pulmón no microcítico	*EGFR*	Mutación T790M	Osimertinib	El genotipado se recomienda en pacientes con cáncer sin tratamiento previo y en caso de resistencia tras tratamientos previos con inhibidores de la tirosina quinasa (TKIs). La detección de fusiones en ctDNA es subóptima y debería de repetirse en el tejido cuando sea posible.
	*EGFR*	Deleciones con desplazamiento del marco de lectura en exón 19, mutación L858R	Erlotinib, Erlotinib + Ramucirumab, Afatinib, Dacomitinib, Gefitinib, Osimertinib, Amivantamab + Lazertinib	
	*EGFR*	Inserciones con desplazamiento del marco de lectura en exón 20 (762_823ins)	Amivantamab	
	*EGFR*	Mutaciones G719, S768I, L861Q	Afatinib	
	*ALK*	Fusiones	Alectinib, Brigatinib, Ceritinib, Crizotinib, Lorlatinib	
	*MET*	D1010, deleción del exón 14, deleciones con desplazamiento del marco de lectura en exón 14, mutaciones de sitios de corte y empalme en exón 14	Capmatinib, Tepotinib	
	*KRAS*	G12C	Sotorasib, Adagrasib	
	*BRAF*	V600E	Dabrafenib + Trametinib, Encorafenib + Binimetinib	
	*RET*	Fusiones	Selpercatinib, Pralsetinib	
	*ROS1*	Fusiones	Crizotinib, Entrectinib, Repotrectinib	
	*NTRK 1/2/3*	Fusiones	Entrectinib, Larotrectinib, Repotrectinib	
	*NTRK 1/2/3*	Mutaciones de resistencia adquirida	Entrectinib, Larotrectinib	
Cáncer colorrectal	*BRAF*	V600E	Encorafenib + Cetuximab	*KRAS/NRAS/BRAF* ^V600E^/MSI para el cáncer colorrectal metastásico sin tratamiento previo, cuando no hay disponibilidad de tejido o se requiere una toma de decisiones terapéuticas urgente. *KRAS/NRAS/BRAF/EGFR*-ECD para pacientes pretratados si se planea un nuevo intento de tratamiento con EGFR.
	*MSI-H*	Inestabilidad de microsatélites alta (MSI-H)	Pembrolizumab, Nivolumab, Ipilimumab + Nivolumab	
	*NTRK 1/2/3*	Fusiones	Entrectinib, Larotrectinib, Repotrectinib	
	*KRAS/NRAS*	Mutaciones del exón 2,3,4	Cetuximab, Panitumumab	
	*KRAS*	G12C	Adagrasib + Cetuximab	
	*ERBB2*	Amplificación	Tucatinib + Trastuzumab	
	*EGFR*	Mutaciones en el dominio extracelular S492, G465, S464, V441	Cetuximab, Panitumumab	
Cáncer pancreático y hepatocelular	MSI-H	Inestabilidad de microsatélites alta (MSI-H)	Pembrolizumab	Cuando no hay disponibilidad de tejido.
	*NTRK 1/2/3*	Fusiones	Entrectinib, Larotrectinib, Repotrectinib	
Cáncer gástrico	*ERBB2*	Amplification	Pembrolizumab + Trastuzumab + Chemotherapy, Trastuzumab + Chemotherapy, Trastuzumab Deruxtecan	Cuando no hay tejido disponible o se necesita una decisión terapéutica urgente.
	MSI-H	Inestabilidad de microsatélites alta (MSI-H)	Pembrolizumab	
	*NTRK 1/2/3*	Fusiones	Entrectinib, Larotrectinib, Repotrectinib	
Cáncer de mama	*PIK3CA*	Mutaciones C420R, E542K, E545A, E545D, E545G, E545K, H1047L, H1047R, H1047Y, Q546E, Q546R	Alpelisib + Fulvestrant, Capivasertib + Fulvestrant	Las mutaciones en *ESR1* deberían analizarse preferentemente en ctDNA. *“*La amplificación de *ERBB2* y las fusiones de *NTRK* cuando no se dispone de tejido.
	*ERBB2*	Amplificación	Ado-Trastuzumab Emtansine, Lapatinib + Capecitabine, Lapatinib + Letrozole, Margetuximab + Chemotherapy, Neratinib, Neratinib + Capecitabine, Trastuzumab, Trastuzumab + Chemotherapy, Trastuzumab + Pertuzumab + Chemotherapy, Trastuzumab + Tucatinib + Capecitabine, Trastuzumab Deruxtecan	
	*ESR1*	D538 y E380, L469V, L536, S463P, Y537	Elacestrant	
	MSI-H	Inestabilidad de microsatélites alta (MSI-H)	Pembrolizumab	
	*NTRK 1/2/3*	Fusiones	Entrectinib, Larotrectinib, Repotrectinib	
Colangiocarcinoma	*IDH1*	Mutaciones R132	Ivosidenib	Cuando no hay tejido disponible o se necesita una decisión terapéutica urgente.
	*FGFR2*	Fusiones	Futibatinib, Pemigatinib	
	MSI-H	Inestabilidad de microsatélites alta (MSI-H)	Pembrolizumab	
	*NTRK 1/2/3*	Fusiones	Entrectinib, Larotrectinib, Repotrectinib	
Cáncer de ovario	*BRCA1/2*	Mutaciones	Olaparib, Olaparib + Bevacizumab, Niraparib, Rucaparib	En mujeres sin variantes patogénicas germinales en *BRCA1/2*, cuando no se dispone de tejido
	MSI-H	Inestabilidad de microsatélites alta (MSI-H)	Pembrolizumab	
Cáncer endometrial	MSI-H	Inestabilidad de microsatélites alta (MSI-H)	Pembrolizumab	Cuando no hay disponibilidad de tejido.
Cáncer de próstata	*BRCA1/2*	Mutaciones	Olaparib, Olaparib + Bevacizumab, Niraparib, Rucaparib	Cuando no hay disponibilidad de tejido.
	MSI-H	Inestabilidad de microsatélites alta (MSI-H)	Pembrolizumab	
Cáncer urotelial	*FGFR*	Mutaciones G370C, R248C, S249C, Y373C	Erdafitinib	Cuando no hay disponibilidad de tejido.
	*FGFR3*	Fusiones	Erdafitinib	
	*NTRK 1/2/3*	Fusiones	Entrectinib, Larotrectinib, Repotrectinib	
Cáncer de tiroides	*BRAF*	V600E	Dabrafenib + Trametinib	Cuando no hay disponibilidad de tejido.
	*RET*	Mutaciones y fusiones	Pralsetinib, Selpercatinib	
	*NTRK 1/2/3*	Fusiones	Entrectinib, Larotrectinib, Repotrectinib	
Sarcoma de tejidos blandos	*NTRK 1/2/3*	Fusiones	Entrectinib, Larotrectinib, Repotrectinib	Cuando no hay disponibilidad de tejido.

^a^Fármacos relacionados, aprobados por la FDA y definidos en OncoKB [120], a fecha de octubre de 2024.

Aparte de los usos de ctDNA actualmente recomendados en la práctica clínica habitual, estudios recientes respaldan otras aplicaciones clínicas en el contexto de enfermedad avanzada. A este respecto, se ha demostrado la utilidad de la fracción tumoral de ctDNA como biomarcador pronóstico independiente en diferentes tipos de cáncer [107]. Del mismo modo, se ha aportado evidencia sobre la eficacia de la caracterización molecular de ctDNA en la selección de candidatos a recibir terapias dirigidas en fases tempranas [108].

### Posibles aplicaciones

A pesar de los avances recientes, existen algunas aplicaciones clínicas de la biopsia líquida que aún no han sido implementadas en la práctica clínica habitual. Tal es el caso del diagnóstico o detección precoz, la detección de enfermedad mínima residual (MRD) y el seguimiento de la enfermedad durante el tratamiento. Aunque las pruebas de ctDNA pueden mejorar los procesos diagnósticos y facilitar la identificación de tumores en estadios tempranos, aún quedan por resolver algunas limitaciones para su implementación en la clínica [4]. Lograr una elevada especifidad y una sensibilidad clínicamente relevante no es sencillo, principalmente debido a que los tumores en estadio temprano liberan niveles reducidos de ctDNA [55]. Para poder implementar las pruebas de ctDNA en la práctica clínica como herramientas de detección validadas, son necesarios más estudios poblacionales a gran escala [4]. De este modo, estudios recientes han aportado evidencia sobre el empleo de ctDNA para el cribado y la detección temprana de pacientes con enfermedades oncológicas [5], 122], 123].

Con respecto a la detección de MRD, el análisis de ctDNA posterior a tratamiento curativo en el cáncer en etapa temprana es un factor predictivo de recurrencia, con una elevada especifidad clínica [124]. En los últimos años, se ha acrecentado el interés en la MRD, manifestado en la realización de múltiples ensayos clínicos aleatorizados basados en el ctDNA en cáncer colorrectal, pulmonar y de mama, en los que se están obteniendo resultados prometedores para la implementación de ctDNA en la evaluación de MRD. En este contexto, se ha demostrado la utilidad de realizar un seguimiento de ctDNA tras cirugía en el cáncer colorrectal resecable, para identificar a los pacientes con alto riesgo de recurrencia y/o mortalidad, con mayor probabilidad de beneficiarse de la quimioterapia adyuvante [66], 125]. Además, el análisis de muestras de ctDNA seriadas en pacientes con cáncer de colon receptores de terapia adyuvante facilita la escalada o desescalada del tratamiento, permitiendo una selección más precisa de aquellos pacientes que verdaderamente se van a beneficiar de la terapia adyuvante, frente al sistema convencional de estadificación de tumores/nódulos/metástasis (TNM) [126]. Cabe señalar que, en un estudio reciente sobre el cáncer de colon localizado, se ha demostrado que la precisión de la predicción de MRD mejora cuando se emplean paneles de NGS que detecten múltiples variantes genéticas de ctDNA en diferentes muestras seriadas de plasma [127]. En el carcinoma pulmonar no microcítico en estadio temprano, se ha observado que la detección de ctDNA residual posterior a tratamiento puede ser un factor predictor de recidiva precoz [128]. Así mismo, en el cáncer de mama, la caracterización molecular de ctDNA puede ser de utilidad para detectar la aparición de recurrencias [129].

El empleo de ctDNA también es prometedor en el seguimiento de la respuesta a tratamiento y el desarrollo de resistencia en pacientes oncológicos [21]. Su corta vida media, sumada a la posibilidad de la toma de muestras en tiempo real, hace del ctDNA una herramienta valiosa para evaluar la dinámica de la enfermedad durante la terapia [4]. Algunos estudios indican una correlación entre los niveles de ctDNA y la respuesta a tratamiento, pudiendo detectar cambios antes que los métodos clínicos tradicionales [21], 130]. Sin embargo, aún no se está implementando el ctDNA en la clínica debido a sus limitaciones, como la necesidad de optimizar las metodologías de la prueba, establecer la frecuencia de seguimiento más adecuada, y a la falta de evidencia suficiente sobre las mejoras en los resultados clínicos [4].

## Comunicación de las variantes genéticas detectadas en ctDNA

La generación de informes sobre los resultados de las pruebas moleculares es crucial a la hora de traducir datos genéticos complejos en información clínica útil. Este tipo de informes deben tener un formato estandarizado, y en ellos debe figurar claramente la fecha de emisión e incluir detalles sobre el diagnóstico, así como información médica relevante, en su caso. Además, los informes deben estar redactados en un lenguaje claro y conciso, y proporcionar información clínicamente relevante de manera comprensible y sencilla. Por otro lado, el formato de estos informes debe permitir su fácil integración en la historia clínica electrónica. La información clínica crítica debe aparecer al principio para su rápida identificación, mientras que los datos de mayor complejidad pueden presentarse de forma simplificada mediante gráficas y tablas [97]. Las alteraciones genéticas se deben describir de manera detallada, incluyendo los genes implicados, el tipo de variantes o características genómicas detectadas (como SNVs, *indels*, CNVs y fusiones), y su impacto esperado en la función proteica. La adopción de una nomenclatura normalizada que se ajuste a la guía de la Human Genome Variation Society (HGVS) (http://varnomen.hgvs.org/) resulta esencial a la hora de evitar confusiones y errores clínicos [94]. En el informe se deben incluir los elementos relevantes para poder realizar un análisis meticuloso y comparaciones longitudinales, como las coordenadas genómicas, la versión del genoma utilizada y la secuencia de referencia del transcrito. La inclusión de la VAF en los informes, siempre que sea posible en las pruebas cuantitativas, proporciona información crucial que permite evaluar la fiabilidad de las variantes detectadas, especialmente en lo relativo al riesgo de falsos negativos.

Siguiendo el sistema de clasificación de la AMP, se recomienda comunicar la presencia de las variantes Tier I a III por orden de relevancia clínica. En términos generales, no se deben incluir las variantes Tier IV, clasificadas como benignas o probablemente benignas. Así mismo, se deben incluir comentarios interpretativos, especialmente en relación a las variantes genéticas de Tier I y II. Las recomendaciones deben estar fundamentadas en la evidencia científica e incluir las citas bibliográficas pertinentes [97]. Las anotaciones sobre aplicabilidad clínica son una parte esencial del informe de resultados, sustentando la interpretación clínica de los resultados. Únicamente se debe evaluar la aplicabilidad clínica de aquellas alteraciones oncogénicas clasificadas como probablemente patogénicas o patogénicas, utilizando marcos de evidencia clínica como ESCAT, el sistema de clasificación OncoKB o la clasificación por niveles de la AMP [131].

Por un lado, cuando no se detecta una variante genética, es preferible emplear términos como “no informativo” o “no detectado” en lugar de “negativo” [4]. En el informe, se debe advertir sobre posibles discrepancias con respecto a los resultados del análisis de tejido tumoral, especialmente cuando no se detecta ninguna variante en el ADN plasmático.

En el análisis de ctDNA, se puede dar el caso de que se identifiquen variantes germinales de manera incidental. En el caso de que se comunicaran dichos hallazgos, sería conveniente diferenciar claramente entre las variantes somáticas y las presuntas variantes germinales, así como incluir información sobre la necesidad de realizar pruebas confirmatorias en leucocitos de sangre periférica, o en otras muestras de tejido normal [78], 79].

Al final del informe, se debe incluir información metodológica y describir las limitaciones del método, detallando las alteraciones que se han incluido en el cribado, el rendimiento de la prueba (como el límite de detección para cada tipo de variantes y la profundidad mínima de secuenciación) y las métricas de calidad más relevantes [86]. Se debe proporcionar información sobre aquellos factores preanalíticos, analíticos o postanalíticos que pudieran influir en la interpretación clínica. Así mismo, es importante mencionar que la sensibilidad de la prueba puede depender de la cantidad de cfDNA empleada. De este modo, cuando se dispone de una cantidad limitada de cfDNA plasmático, se puede ajustar la sensibilidad descrita o incluir una nota de advertencia en el informe [86].

## Perspectivas futuras del ctDNA

Además del estudio de las variantes genéticas, una de las áreas de investigación sobre ctDNA más prometedoras es la epigenómica (metilación del ADN) y la fragmentómica. Se ha demostrado la utilidad de estos campos para la detección precoz del cáncer, la identificación del origen del tumor, y la evaluación de la respuesta a terapia [5], 35]. También es importante señalar que la inteligencia artificial, especialmente los algoritmos de aprendizaje automático, está adquiriendo un papel cada vez más relevante en el descubrimiento e implementación de nuevos biomarcadores de ctDNA. Además de permitir análisis más precisos y completos de la genómica, epigenómica y fragmentómica del cáncer [132], la inteligencia artificial también facilita la integración de estas “ómicas” con los datos clínicos, impulsando el avance de la oncología médica personalizada [133].

A pesar de estos avances, aún quedan varios aspectos por resolver, antes de poder integrar el análisis de ctDNA en la práctica clínica habitual. La principal limitación es la sensibilidad, especialmente en el cáncer en etapa temprana, cuando los niveles de ctDNA suelen ser bajos. Es necesario intensificar las investigaciones para mejorar la sensibilidad de las pruebas de ctDNA a través de la NGS y la dPCR [3], 134].

Una ventaja importante del análisis del ctDNA es su capacidad para proporcionar información en tiempo real sobre la heterogeneidad del tumor. Al capturar las variaciones genómicas espaciales y temporales en un paciente, el ctDNA puede ofrecer una imagen más completa de la evolución del tumor que las biopsias de tejido tradicionales. Esto resulta de especial utilidad en los estadios más avanzados del cáncer, cuando los tumores suelen mostrar una heterogeneidad considerable, lo que contribuye a desarrollar resistencia al tratamiento [4], 124]. Por otro lado, los falsos positivos siguen siendo un problema a resolver, más si cabe cuando las mutaciones en el ctDNA se solapan con variantes CHIP [75], lo que evidencia la necesidad de desarrollar y validar pruebas más sólidas.

El análisis del ctDNA también tiene aplicaciones clínicas para la detección de MRD. Monitorizar los niveles de ctDNA podría ayudar a identificar a los pacientes con mayor riesgo de recidiva, permitiendo así una intervención terapéutica temprana [4]. Presumiblemente, los ensayos clínicos actualmente en curso proporcionarán datos clínicos cruciales sobre el papel del ctDNA en la MRD, el seguimiento de la evolución del tumor, y para orientar las decisiones terapéuticas [66].

Para mejorar la utilidad diagnóstica del ctDNA, resulta esencial estandarizar los procedimientos preanalíticos y analíticos. Así, se están llevando a cabo iniciativas, como el BloodPac en los Estados Unidos y el Cancer-ID en Europa, destinadas a establecer procedimientos operativos estándar para el análisis de ctDNA [135], 136]. La normalización no solo mejorará la reproducibilidad, sino que también facilitará la implementación clínica a gran escala del análisis de ctDNA.

Otro prometedor campo de investigación es la combinación del análisis de ctDNA con otros biomarcadores circulantes, como las células tumorales circulantes (CTCs) y las vesículas extracelulares. Esta estrategia podría arrojar información más completa sobre la biología del tumor, ayudando a perfeccionar el diagnóstico y orientar las decisiones terapéuticas [1]. Por otro lado, existe un interés creciente en el estudio del ctDNA en otros fluidos biológicos diferentes al plasma, por su potencial para mejorar el manejo de las enfermedades oncológicas en algunos tipos de tumores [46], 52], 137].

A pesar de todos estos avances, la implementación del análisis de ctDNA en la práctica clínica sigue siendo limitada debido a ciertas dificultades técnicas y económicas. Los métodos basados en NGS, aunque cuentan con una elevada sensibilidad, precisan el empleo de un equipamiento de laboratorio muy sofisticado y requieren tiempo, lo que dificulta su implementación a gran escala [138]. Para superar estas limitaciones, es esencial seguir desarrollando estas tecnologías y diseñar pruebas más sencillas y con una mejor relación coste-beneficio [3].

## Conclusiones

El análisis de ctDNA está abriendo nuevos horizontes hacia un manejo más personalizado de las enfermedades oncológicas. En la última década, se han logrado importantes avances en las tecnologías de análisis de ctDNA, y numerosos estudios demuestran que esta técnica podría revolucionar el abordaje de los pacientes oncológicos [1]. Para los pacientes con cáncer avanzado, las pruebas de ctDNA validadas y con sensibilidad adecuada son de utilidad para identificar mutaciones accionables con terapias dirigidas, pudiendo ser empleadas en la práctica clínica, especialmente cuando urge disponer de los resultados, o cuando no se puede o no se debe realizar una biopsia de tejido. Además, el análisis de ctDNA presenta un gran potencial en el diagnóstico del cáncer, la detección de MRD, y el seguimiento y la evaluación de la respuesta a terapia [4].

En conclusión, el análisis de ctDNA supone una gran oportunidad para avanzar en la detección temprana del cáncer y desarrollar tratamientos personalizados, lo que mejorará significativamente los resultados clínicos. No obstante, para poder impulsar su implementación clínica generalizada, aún es necesario validar y normalizar la técnica y reducir costes. A medida que los ensayos clínicos en curso vayan arrojando datos relevantes, es probable que el ctDNA, empleado en combinación con otros biomarcadores circulantes, acabe convirtiéndose en una piedra angular de los laboratorios clínicos.
